# Low Birth Weight in Perinatally HIV-Exposed Uninfected Infants: Observations in Urban Settings in Cameroon

**DOI:** 10.1371/journal.pone.0093554

**Published:** 2014-04-03

**Authors:** Casimir Ledoux Sofeu, Josiane Warszawski, Francis Ateba Ndongo, Ida Calixte Penda, Suzie Tetang Ndiang, Georgette Guemkam, Nicaise Makwet, Félicité Owona, Anfumbom Kfutwah, Patrice Tchendjou, Gaëtan Texier, Maurice Tchuente, Albert Faye, Mathurin Cyrille Tejiokem, The ANRS-PEDIACAM study group

**Affiliations:** 1 Service d’Epidémiologie et de Santé Publique, Centre Pasteur du Cameroun, Membre du Réseau International des Instituts Pasteur, Yaoundé, Cameroun; 2 Université de Yaoundé I, IRD UMI 209 UMMISCO, Yaoundé, Cameroun; 3 Laboratoire International en Recherche Informatique et Mathématiques Appliquées, Equipe Idasco, Yaoundé, Cameroun; 4 Equipe 4 (VIH et IST) - INSERM U1018 (CESP), Le Kremlin Bicêtre, France; 5 Assistance Publique des Hôpitaux de Paris, Service d’Epidémiologie et de Santé Publique, Hôpital de Bicêtre, Le Kremlin Bicêtre, France; 6 Université de Paris Sud 11, Paris, France; 7 Centre Mère et Enfant de la Fondation Chantal Biya, Yaoundé, Cameroun; 8 Hôpital de Jour, Hôpital Laquintinie, Douala, Cameroun; 9 Faculté de Médecine et des Sciences Pharmaceutiques, Université de Douala, Douala, Cameroun; 10 Service de Pédiatrie, Centre Hospitalier d’Essos, Yaoundé, Cameroun; 11 Maternité Principale, Hôpital Central, Yaoundé, Cameroun; 12 Service de Virologie, Centre Pasteur du Cameroun, Membre du Réseau International des Instituts Pasteur, Yaoundé, Cameroun; 13 SESSTIM (UMR 912), Université Aix-Marseille, Marseille, France; 14 Assistance Publique des Hôpitaux de Paris, Pédiatrie Générale, Hôpital Robert Debré, Paris, France; 15 Université Paris 7 Denis Diderot, Paris Sorbonne Cité, Paris, France; University of British Columbia, Canada

## Abstract

**Background:**

The consequences of maternal HIV infection for fetal growth are controversial. Here, we estimated the frequency of small for gestational age and gender (SGAG) among neonates born to HIV-infected or uninfected mothers and assessed the contribution, if any, of maternal HIV to the risk of SGAG.

**Methods:**

The data used were obtained from the ANRS-Pediacam cohort in Cameroon. Pairs of newborns, one to a HIV-infected mother and the other to an uninfected mother, were identified during the first week of life, and matched on gender and recruitment site from 2007–2010. SGAG was defined in line with international recommendations as a birth weight Z-score adjusted for gestational age at delivery and gender more than two standard deviations below the mean (−2SD). Considering the matched design, logistic regression modeling was adjusted on site and gender to explore the effect of perinatal HIV exposure on SGAG.

**Results:**

Among the 4104 mother-infant pairs originally enrolled, no data on birth weight and/or gestational age were available for 108; also, 259 were twins and were excluded. Of the remaining 3737 mother-infant pairs, the frequency of SGAG was 5.3% (95%CI: 4.6–6.0), and was significantly higher among HIV-infected infants (22.4% vs. 6.3%; p<.001) and lower among HIV-unexposed uninfected infants (3.5% vs. 6.3%; p<.001) than among HIV-exposed uninfected infants. Similarly, SGAG was significantly more frequent among HIV-infected infants (aOR: 4.1; 2.0–8.1) and less frequent among HIV-unexposed uninfected infants (aOR: 0.5; 0.4–0.8) than among HIV-exposed uninfected infants. Primiparity (aOR: 1.9; 1.3–2.7) and the presence of any disease during pregnancy (aOR: 1.4; 1.0–2.0) were identified as other contributors to SGAG.

**Conclusion:**

Maternal HIV infection was independently associated with SGAG for HIV-exposed uninfected infants. This provides further evidence of the need for adapted monitoring of pregnancy in HIV-infected women, especially if they are symptomatic, to minimize additional risk factors for SGAG.

## Introduction

Impaired fetal growth can have devastating effects on subsequent infant growth, survival [1]–[9] and neurocognitive development [10]. Fetal growth is influenced by diverse medical, obstetrical, socio-economic and behavioral factors [11]–[16]. Studies in both industrialized and developing countries report inconsistent results concerning the consequences of maternal HIV infection on fetal growth. Some authors report that anthropometric measures for both HIV-infected and uninfected infants born to HIV-infected mothers are significantly lower than those for infants born to HIV-negative mothers [1], [4], [13], [17]. Other studies failed to detect any maternal HIV-related effects on growth [3], [15], [16], [18], [19]. The reported effects on fetal growth of antiretroviral therapy used during pregnancy are similarly contradictory [20]–[23]. There is also conflicting data regarding perinatal HIV-exposure and post natal infant growth. Some authors report no differences whereas others have observed growth faltering in HIV-exposed uninfected infants [4], [10], [18], [19], [24], [25]. These discrepancies may be the consequence of methodological issues, and in particular the use in most of these studies of anthropometric statistics that do not take into account both gestational age at delivery and gender.

Programs to prevent mother-to-child HIV transmission have been implemented and are generally successful in sub-Saharan Africa: they include improved healthcare coverage and access to antiretroviral therapy for HIV-infected women and their infants. As a result, most infants now escape HIV infection and the number of HIV-exposed uninfected infants will increase such that any problems specifically associated with this group will become of major public health importance. Therefore, data on HIV-exposed uninfected infant growth are needed as a basis for preventive strategies to ensure appropriate care and to evaluate the impact of HIV and antiretroviral drug use on infant life [4], [24], [25].

We estimated the frequency of SGAG among HIV-infected and uninfected newborns to HIV-infected mothers, and compared these values to those for newborns to uninfected mothers, in urban settings of Cameroon. We used data from the ongoing ANRS-Pediacam study, in which infants were enrolled at birth or before the 8^th^ day of life. We studied birth weight z-scores adjusted on gestational age at delivery and gender to appreciate the effects of maternal HIV infection on the risk of SGAG in HIV-exposed uninfected infants.

## Methods

### Ethics Statement

The ANRS-Pediacam study was granted ethical approval in Cameroon by the National Ethics Committee and in France by the Biomedical Research Committee of the Pasteur Institute of Paris. The Cameroon Ministry of Public Health gave administrative authorization to start the study. Written informed consent was obtained from parents or guardians prior to inclusion of infants into the research project.

### Data Source

Data used in this analysis were obtained from the ANRS-Pediacam cohort based in three referral hospitals in Cameroon (The Maternity of the Central hospital/Mother and Child Center of the Chantal Biya Foundation (MCH/MCC-CBF), Essos Hospital Center in Yaoundé (EHC) and the Laquintinie Hospital in Douala (LH)) and coordinated by the Centre Pasteur of Cameroon. The ANRS-Pediacam cohort study is observational and was designed to assess the feasibility of early infant diagnosis of HIV and early antiretroviral multitherapy in HIV-infected infants, and to evaluate the humoral response of these children to vaccines of the Expanded Program on Immunization [26]. All HIV-infected mothers who were seen from November 2007 to October 2010 at one of the participating maternity or pediatric wards during the first week after delivery were invited to participate in the study. Infants born to uninfected mothers matched on study site and sex were also identified and enrolled during the first week of life as controls. The newborn pairs were followed, according to the Cameroon National EPI calendar, at 6, 10 and 14 weeks. Samples for HIV virological testing were collected from HIV-exposed infants at the first follow-up visit planned at 6 weeks, as previously described [26]. Incentives, including free medical support for consultation, biological analysis, additional vaccines and reimbursement of transport costs, were provided to parents/caregivers by the project during follow up visits.

### Participants

**Figure 1:**
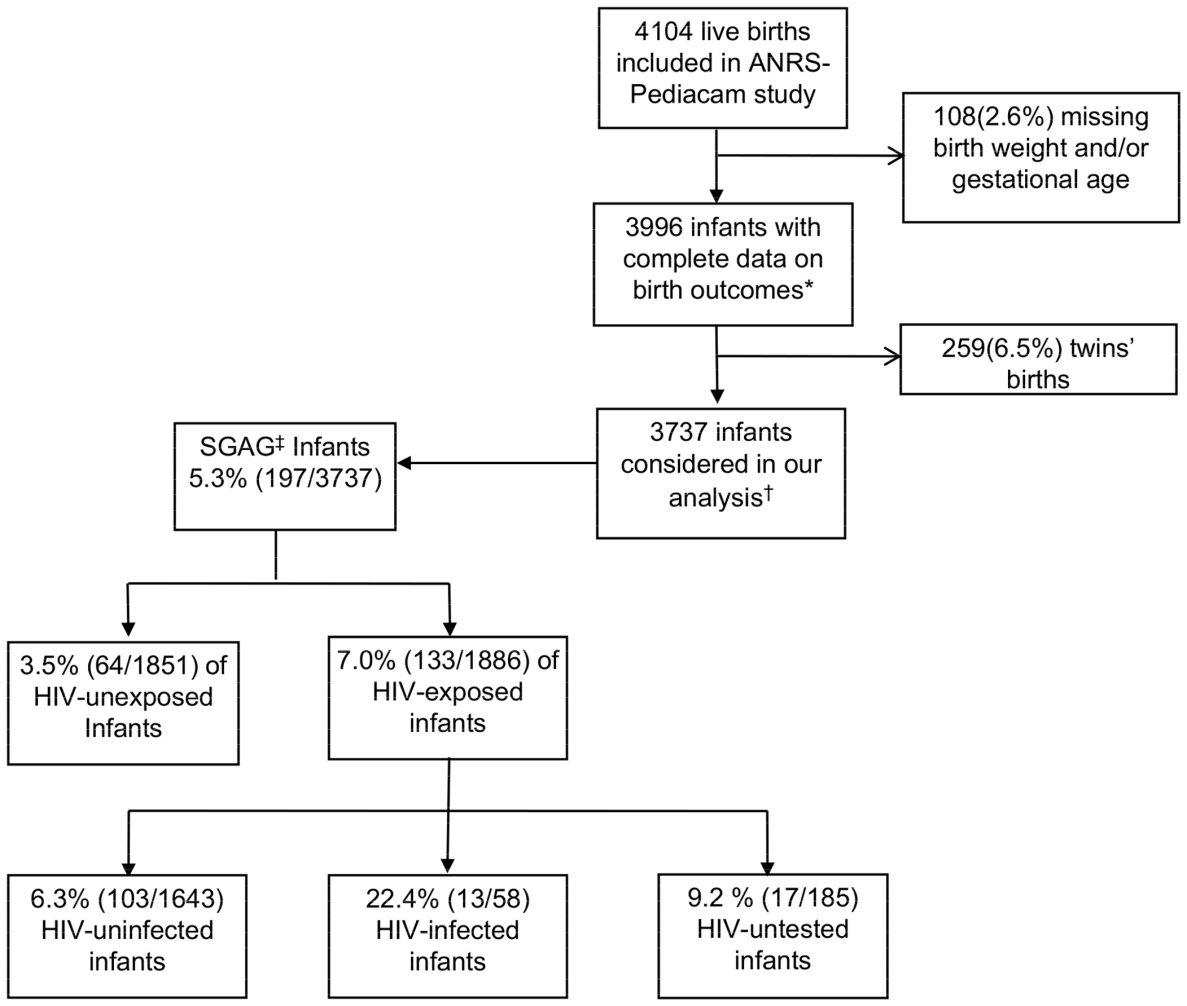
Study population and frequency of SGAG, ANRS 12140-Pediacam study, Cameroon, 2007–2010. *Birth outcomes refer to birth weight and gestational age. ^†^ 9.3% (346) of infants were excluded from the multivariate analysis because of data for potentially associated factors were missing. ^‡^SGAG: small for gestational age and gender.

Among the 4104 mother-infant pairs initially enrolled in the ANRS-Pediacam study, birth weight and/or gestational age data were missing for 108; these infants were excluded because of the lack of adequate outcome data. An additional 259 cases involved twin, and because multiple birth increases the risk of low birth weight, these cases were also excluded from the study [7]. Our analyses were thus conducted on the remaining 3737 mother-infant pairs (Figure 1).

### Data Collection

Data were collected by interviewing the mothers/guardians and completed using the mothers’ medical records (in their hospital books). Socioeconomic and demographic characteristics collected included: mothers’ age, education level, employment and marital status, electricity and water supply at home, existence of a functional fridge at home. The obstetrical history recorded included: gravidity, use of malarial drugs and pathologies during pregnancy, date and mode of delivery, gestational age at delivery, infant birth weight, length, head circumference and gender, and any neonatal or post partum complications. The medical data included: HIV status according to the Cameroonian national algorithm based on rapid tests, and the use of any ART for PMTCT or for the mothers’ own health during pregnancy (ART initiated only at delivery was not considered). During the recruitment period of this study, the national PMTCT policy in Cameroon was based on the 2006 WHO guidelines: pregnant HIV-infected women who had CD4 cell counts of 200 cells/mm^3^ or less were advised to start ART for their own health, and to continue lifelong ART using a triple combination regimen; however, women with higher CD4 cell counts were not considered medically eligible for such treatment, and were advised to take zidovudine (AZT) from 28 weeks of gestation plus a single-dose nevirapine at the onset of labor, a combination of AZT+lamivudine (3TC) during delivery and one week postpartum, with infant prophylaxis for one week after birth [27].

The obstetrical estimate of gestational age was mainly based on the last menstrual period unless adjusted by the midwife on the basis of clinical assessment in conformity with routine practice. These estimations were sometimes confirmed by obstetrical ultrasound scans. Infant birth weight (in grams) was measured in the recumbent position using a Salter scale. For babies delivered at home or on the way to the maternity, weight measured at arrival was recorded. Birth length was measured with the baby supine and the crown of the head touching a vertical headboard. The legs were extended gently and the length recorded using a tape graduated in centimeters from the headboard to the infant’s heel. Head circumference (occipitofrontal) was also measured using a tape positioned just above the eyebrows and placed posteriorly.

### Outcome and Main Exposure Definitions

The studied outcome was SGAG (small for gestational age and gender) defined as a birth weight Z-score adjusted for gestational age at delivery and gender that is more than two standard deviations below the mean (−2SD), in line with international recommendations [28]. Birth weight Z-scores were calculated using French standards, calculated from AUDIPOG [29]. The AUDIPOG database include data from 211,337 infants born in 209 French maternities between 1999 and 2005. Based on these data, different model equations with high order polynomials have been fitted to calculate predicted mean births weight and standard deviations for male and female infants born at any gestational age. In this study, we used these equations to generate Z-scores using the following formula: Z score = (WO–WP)/SDP, where WO: weight observed, WP: weight predicted, SDP: standard deviation predicted. The main exposure investigated was the HIV status of the neonate, classified into three categories: HIV-infected, HIV-uninfected exposed to maternal HIV, HIV-uninfected born to a HIV seronegative mother. The HIV status of infants born to HIV-infected mothers was determined at age 6 weeks by a polymerase chain reaction (PCR)-based test. If HIV was not detected, the infant was considered to be uninfected with HIV. Infants were considered to be HIV-infected if the first and the second sample were positive. Infants infected by HIV subsequently through breastfeeding were considered to be HIV-uninfected but exposed at the time of this analysis.

### Statistical Analysis

Maternal and infant characteristics were described using frequencies for categorical variables, medians and interquartile ranges for continuous variables, and were compared between the three groups of infants defined according to their HIV status. The first step was to analyze the association between the risk of SGAG and the infant HIV status (three main categories), with each sociodemographic, medical and obstetrical characteristic separately, to identify potential confounders. The independent association of SGAG with maternal and infant HIV status, was studied in the second step, taking into account potential confounders. Logistic regression models, all adjusted for matching variables (study site and infant’s gender), were used for both these steps. In the second step, the initial model included variables known to be associated with SGAG, or found to be significant at the 0.25 level in first step of the analysis. The final model was obtained by successively removing variables not associated at a p-value <0.05 only if the odds ratios for remaining variables were unchanged and taking interactions into account. Known risk factors were maintained in the final model [30]. The above analyses were also conducted separately for HIV-uninfected infants born to HIV-negative mothers and HIV-exposed uninfected infants. All analyses were performed using R version 2.12.1 software and Epicalc package. A p-value of 0.05 was considered as the cutoff for statistical significance.

## Results

### Study Population Description

Among the 3737 mother-infant pairs included in this analysis (Figure 1), 1886 infants were born to HIV-infected mothers and 1851 born to HIV-uninfected mothers; 45.8% (1713) were included at the Central Hospital Maternity/Mother to Child Center of the Chantal Biya Foundation (CHM/MCC-CBF), 25.4% (951) at the Hospital Center Essos (HCE) in Yaoundé and 28.7% (1073) at the Laquintinie Hospital (LH) in Douala. About 85.5% (3196) of all the infants enrolled were born in the three maternities participating in the study, 13.8% (514) in other maternities of the cities and 0.7% (27) at home or on their way to a healthcare facility.

The median age at inclusion of infants was 2 days (IQR: 1–5) for infants born at hospital, and 4 days for those born at home or on their way to hospital (IQR: 2–6). The two groups of infants were comparable regarding anthropometric characteristics but there were other significant differences: the mothers of infants born at home had a lower level of education (40.7% vs. 13.6%, p = .001), and more of the infants were born before term (<37 weeks) (37.0% vs. 11.3%, p<.001). The median age of mothers was 29.0 years (25.0–33.0), and this value was significantly lower for HIV-uninfected mothers than HIV-infected mothers: (28.0 years (24.0–32.0) vs. 29.5 years (26.0–33.0); p<.001). The median gestational age at delivery was 39.0 (IQR: 38.0–40.0) weeks.

About 89.3% (1685/1886) of HIV-infected mothers received ART before or during pregnancy for PMTCT and/or for their own health. Complete data on ART regimen and time at first exposure was available for 1553 of these: 792 (51.0%) took ART for prophylaxis, 331 (21.3%) started HAART during pregnancy and 430 (27.7%) before pregnancy. Nearly half of the HIV-infected mothers (44.9% (846/1886)) knew their HIV status before pregnancy. Only 928 mothers reported CD4 counts determined during pregnancy. Of the 1886 infants born to HIV-infected mothers, 1701 were tested for HIV at a median age of 6.0 weeks (IQR: 6.0–7.0) and 58 were infected.

Maternal and infant characteristics at delivery for the HIV-exposed uninfected infant group were significantly different from those for the HIV-unexposed uninfected infant group but similar to those for the HIV-infected infant group (Table 1). Relative to the HIV-unexposed uninfected infant group, mothers of the HIV-exposed uninfected infant group were mostly of low socio-demographic and economic background (low level of education, absence of running water at home, and no functional fridge at home). The proportion of primiparous women was lower among mothers of HIV-exposed uninfected infants than mothers of HIV-unexposed uninfected infants (14.8 % vs. 30.3%, p<0.001) and similar for the HIV-infected group. The proportion of premature births was similar in the three groups.

**Table 1:** Comparison of the characteristics of mothers and neonates according to infant HIV status in the ANRS 12140-Pediacam study, Cameroon, 2007–2010.

	HIV-infected infants			HIV-exposed uninfected infants			HIV-unexposed uninfected infants		
	N = 58			N = 1643			N = 1851		
Mother and neonate characteristics		n	%		n	%		n	%
**Birth outside a study site**	58			1643			1851		
Yes		22	37.9^ ‡‡^		411	25.0		62	3.3^**^
**Mother’s age at delivery**	58			1641			1849		
> 34 years		6	10.3 ??		282	17.2		245	13.2^**^
21–34years		48	82.8		1303	79.4		1440	77.9
< 21 years		4	6.9		56	3.4		164	8.9
**Mother’s level of education**	57			1632			1830		
Higher education		6	10.5 ??		285	17.5		573	31.3^**^
Secondary		42	73.7		1053	64.5		1087	59.4
None or primary education		9	15.8		294	18.0		170	9.3
**Maternal marital status**	57			1636			1836		
Married		9	15.8 ??		405	24.8		690	37.6^**^
Cohabitation		25	43.9		730	44.6		598	32.6
Single/divorced/widow		23	40.3		501	30.6		548	29.8
**Water supply at home**	56			1620			1813		
Yes		30	53.6 ??		885	54.6		1246	68.7^**^
**Functional fridge at home**	55			1617			1805		
Yes		30	54.5 ??		875	54.1		1320	73.1^**^
**Any disease during pregnancy**	56			1612			1803		
Yes		26	46.4 ??		643	39.9		578	32.1^**^
**Mode of delivery**	58			1643			1851		
Caesarean section		3	5.2		146	8.9		211	11.4
**Parity**	58			1639			1837		
primiparous		13	22.4??		242	14.8		557	30.3^**^
**Antiretroviral during pregnancy**	58			1632					
Yes		41	70.7**		1508	92.4			
**Low birth weight**	58			1643			1851		
Yes <2500 g		11	19.0^††^		123	7.5		85	4.6**
**Preterm birth**	58			1643			1851		
Yes (<37 weeks)		10	17.2 ??		200	12.2		191	10.3 ??
**Hypotrophy** *	48			1443			1660		
Yes		6	12.5^ ‡‡^		64	4.4		46	2.8^‡‡^
**SGAG** †	58			1643			1851		
Yes		13	22.4**		103	6.3		64	3.5**
**height Z-score<**−**2SD** ‡	51			1562			1733		
Yes		3	5.9 ??		107	6.8		47	2.7**
**Head circumference**	53			1556			1714		
Z-score<−2SD		4	7.5 ??		87	5.6		63	3.7^‡‡^

*Hypotrophy defined as low birth weight (<2500 g) in infants born at term (gestational age > = 37 weeks).

† SGAG: weight Z-score more than 2 standard deviations below the mean

‡ SD: standard deviation.

p-value using “HIV-exposed uninfected group” as the reference: ** p <.001 ^††^.001 ≤ p <.01 ^‡‡^.01 ≤ p <.05.

$$ .05 ≤ p ≤.10.

?? p >.10.

### Infants Anthropometric Characteristics and Frequency of SGAG

The median birth weight was 3,250 g (IQR: 2,910–3,500), significantly higher in infants born to HIV-uninfected mothers than HIV-exposed uninfected infants (3,300 g vs. 3,200 g; p<.001) and lower in HIV-infected infants than HIV-exposed uninfected infants (2,960 g vs. 3,200 g; p<.001). The length at birth was 50 cm (IQR: 49–52) and head circumference 34 cm (IQR: 33–35).

HIV-exposed uninfected infants had lower mean birth weight Z-score (−0.13 *vs.* 0.11; p<.001), lower mean length Z-score (0.39 *vs.* 0.78; p<.001) and lower mean head circumference Z-score (0.07 *vs.* 0.16; p = .002) than HIV-unexposed uninfected infants. HIV-exposed uninfected infants had higher mean birth weight Z-score (−0.13 *vs.* −0.67; p<.04) than HIV-infected infants, and comparable mean length Z-score (0.39 *vs.* 0.10; p<.16) and mean head circumference Z-score (0.07 *vs.* 0.44; p = .60).

The overall proportion of low birth weight was 7.5% among HIV-exposed uninfected infants and 4.4% of them were hypotrophic. These proportions were significantly higher than for the HIV-unexposed uninfected infant group (4.6% and 2.8%, respectively) and lower than for the HIV-infected infant group (19.0% and 12.5%, respectively) (Table 1).

The overall frequency of SGAG was 5.3% [CI: 4.6–6.0], and SGAG was significantly more frequent among HIV-exposed infants than HIV-unexposed uninfected infants (7.0% vs 3.5%; p = <.001, Figure 1). The frequency of SGAG was 22.4% [11.7–33.1] among the HIV-infected infants, 6.3% [5.1–7.5] among the HIV-exposed uninfected infants, 3.5% [2.7–4.3] among the HIV-unexposed uninfected infants, and 9.2% [5.0–13.4] among HIV-exposed untested infants (Figure 1 and Table 1).

### Factors Associated with SGAG

In the first step of the analysis, the association between covariates and SGAG adjusted for study site and infant’s gender were investigated (Table 2): exposure to maternal HIV infection, primiparity, birth outside of a study site, any maternal illness during pregnancy, low maternal education level, and lack of a functional fridge at home were each significantly associated with SGAG. The use of anti-malaria drugs and the marital status of mothers tended to be associated with SGAG.

**Table 2:** Relation between characteristics of mother-infant pairs and small for gestational age and gender (SGAG) in infants, ANRS 12140-Pediacam study, Cameroon, 2007–2010.

	Analysis adjusted on study site and gender*				
	SGAG†				
Mother and neonate characteristics	N	n (%)	aOR	95%CI	p-value
**Infant status** (n = 3552)					
HIV-infected	58	13 (22.4)	4.5	(2.3–8.7)	<.001
HIV-uninfected exposed to maternal HIV	1643	103 (6.3)	1 (ref)		
HIV-uninfected unexposed to maternal HIV	1851	64 (3.5)	0.5	(0.4–0.7)	
**Birth outside a study site** (n = 3737)					
No	3196	151 (4.7)	1 (ref)		<.001
Yes	541	46 (8.5)	1.9	(1.3–2.6)	
**Mother’s level of education** (n = 3703)					
None or primary education	512	38 (7.4)	1.7	(1.1–2.8)	.05
Higher education	890	41 (4.6)	1 (ref)		
Secondary	2301	116 (5.0)	1.2	(0.8–1.7)	
**Mother’s age at delivery** (n = 3732)					
> 34 years	556	28 (5.0)	0.9	(0.6–1.4)	.76
21–34years	2942	156 (5.3)	1 (ref)		
< 21 years	234	10 (4.3)	0.8	(0.4–1.5)	
**Maternal marital status** (n = 3713)					
Cohabitation	1436	78 (5.4)	1.3	(0.9–1.9)	.07
Married	1145	48 (4.2)	1 (ref)		
Single/divorced/widow	1132	71 (6.3)	1.6	(1.1–2.3)	
**Water supply at home** (n = 3672)					
Yes	2263	117 (5.2)	1 (ref)		.55
No	1409	75 (5.3)	1.1	(0.8–1.5)	
**Functional fridge at home** (n = 3660)					
Yes	2325	105 (4.5)	1 (ref)		.01
No	1335	87 (6.5)	1.5	(1.1–2.0)	
**Use of anti-malarial drugs during pregnancy** (n = 3695)					
Yes	3406	174 (5.1)	1 (ref)		.12
No	289	21 (7.3)	1.5	(0.9–2.3)	
**Any maternal disease during pregnancy** (n = 3653)					
No	2347	110 (4.7)	1 (ref)		.01
Yes	1306	83 (6.4)	1.5	(1.1–2.0)	
**Mode of delivery** (n = 3737)					
Vaginal	3344	174 (5.2)	1 (ref)		.48
Caesarean section	393	23 (5.9)	1.2	(0.8–1.8)	
**Parity** (n = 3719)					
Multiparous	2872	139 (4.8)	1 (ref)		.02
Primiparous	847	58 (6.8)	1.5	(1.1–2.0)	

*Analysis was adjusted on infant gender and recruitment site which were used to match data (logistic regression).

† Weighted Z-score adjusted on gestational age and gender more than 2 standard deviation below the mean.

aOR: adjusted odds ratio; CI: Confidence Interval.

In multivariate modeling (Table 3), some of the independent predictors of SGAG were similar to those suggested in the first step analysis; in particular, exposure to maternal HIV infection appeared to be an important factor. Relative to HIV-exposed uninfected infants (reference group), the odds of SGAG were fourfold higher for infants who were HIV-infected (aOR: 4.0; 2.0–8.1) and half for infants who were HIV-unexposed uninfected (aOR: 0.5; 0.4–0.8). Other significant contributors to SGAG identified were primiparity (aOR: 1.9; 1.3–2.7), and the existence of any maternal disease during pregnancy (aOR: 1.4; 1.0−2.0); the odds of SGAG also tended to be higher for those infants with no functional fridge at home (aOR: 1.3; 0.9–1.9). No significant interactions were identified between the principal variable of interest (mother-infant HIV status) and other independent variables included in the model or between certain independent variables.

**Table 3:** Maternal HIV status and small for gestational age and gender (SGAG) in infants considering other maternal and infant characteristics, multivariate analysis; ANRS-Pediacam study, Cameroon, 2007−2010.

N = 3342	IUGR†		
Maternal and neonate characteristics	Adj. OR	95%CI	p-value
**Infant status**			
HIV-infected	4.0	(2.0–8.1)	<.001
HIV-uninfected exposed to maternal HIV	1 (ref)		
HIV-uninfected not exposed to maternal HIV	0.5	(0.4–0.8)	
**Clinical site** *			
EHC $	1.4	(0.9–2.1)	.1
MCH/MCC-CBF ‡	1 (ref)		
LH ^?^	0.9	(0.6–1.3)	
**Sex** *			
Female	1 (ref)		.02
Male	1.4	(1.0–2.0)	
**Birth outside a study site**			
No	1 (ref)		.12
Yes	1.4	(0.9–2.1)	
**Mother’s level of education**			
None or primary education	1.5	(0.9–2.6)	.16
Higher education	1 (ref)		
Secondary education	1.0	(0.7–1.6)	
**Mother’s age at delivery**			
> 34 years	1.1	(0.7–1.7)	.59
21−34years	1 (ref)		
< 21 years	0.8	(0.4–1.6)	
**Maternal marital status**			
Cohabitation	0.9	(0.6–1.3)	.77
Married	1 (ref)		
Single/divorced/widow	1.1	(0.8–1.7)	
**Functional fridge at home**			
Yes	1 (ref)		.08
No	1.3	(0.9–1.9)	
**Using of anti-malarial drugs during pregnancy**			
Yes	1 (ref)		.58
No	1.2	(0.7–2.0)	
**Any disease during pregnancy**			
No	1 (ref)		.03
Yes	1.4	(1.0–2.0)	
**Mode of delivery**			
Vaginal	1 (ref)		.15
Caesarean section	1.5	(0.9–2.4)	
**Parity**			
Multiparous	1 (ref)		<.001
primiparous	1.9	(1.3–2.7)	

*Weighted Z-score adjusted on gestational age and sex of newborn more than 2 standard deviations below the mean.

† CHM/MCC-CBF: Central hospital Maternity/Mother and Child Center of the Chantal Biya Foundation, Yaoundé.

‡ EHC: Essos Hospital Center, Yaoundé.

$ LH: Laquintinie Hospital, Douala.

Adj. OR: adjusted Odd ratio; CI: Confidence Interval.

Subgroup analyses (not shown) were conducted and indicated that within the HIV-unexposed uninfected infant group, primiparity (aOR 2.1, 1.1–3.7, p = .02) was associated with SGAG. Such association was also found for the HIV-exposed uninfected infant group (aOR 2.1, 1.1−4.3, p = .05). Among HIV-exposed uninfected infants, the use of any antiretroviral drugs during pregnancy (aOR 0.8, 0.1−6.5, p = .82) was not associated with SGAG. Using the absence of a functional fridge at home as a proxy of socioeconomic status, no independent relation was found either in HIV-exposed uninfected or in HIV-unexposed uninfected infant groups.

Univariate analysis was conducted for HIV-exposed infant group, taking various measures of HIV severity into account, and in particular CD4 lymphocyte count and ART status of HIV-infected mothers; no relation between ART started before or during pregnancy and SGAG was found; however, despite the small sample size (n = 845) in this multivariate analysis, severe immunosuppression in HIV-infected pregnant women (CD4 count <200 cells/mm3) was associated with SGAG (aOR: 2.5; IC: 1.3−4.8).

## Discussion

We investigated the effect of maternal HIV infection on standardized birth weight using data from the ANRS-PEDIACAM study collected in three referral hospitals in the two biggest cities of Cameroon. Almost all HIV-infected mothers at these hospitals were contacted, and the study enrollment rate was 85% (data not shown). The use of ART prophylaxis to reduce vertical transmission is becoming a standard in these hospitals, and indeed about 90% of HIV-infected mothers included in our study reported the use of antiretroviral drugs (combinations or single drugs) during pregnancy or at delivery.

Our main result we report is the strong association between maternal HIV infection and both SGAG and infant HIV status. Overall, the proportion of SGAG was 3.5% among HIV-unexposed uninfected infants, twice as high (6.3%) among HIV-exposed uninfected infants and nearly seven times higher (22.4%) among HIV-infected infants. Compared to HIV-exposed uninfected infants, and taking into account several potential confounders (likely to be more frequent along HIV-exposed infants, Table 1), SGAG was significantly more frequent among HIV-infected infants and less frequent among HIV-unexposed uninfected infants. Also, we observed that HIV-unexposed uninfected infants had higher birth weight Z-scores and HIV-infected infants had lower birth weight Z-scores than HIV-exposed uninfected infants.

The comparison of our results with those of other studies is complicated for various reasons: differences in the populations studied, the availability of ART at the time of the study, socioeconomic environments, sample sizes and the outcome criteria used to assess growth up to birth. Nevertheless, our data lead to conclusions consistent with the findings of studies which report significant differences in birth weights between HIV-infected infants, and HIV-exposed uninfected infants and/or HIV-exposed uninfected and HIV-unexposed uninfected infants [1], [4], [13], [17]. Nevertheless, some studies found no difference in birth weight between these groups of infants [3], [15], [16], [18], [19].

We observed that HIV-infected infants were smaller at birth than HIV-exposed uninfected infants. This suggests a direct effect of maternal HIV on fetal growth but the mechanisms of any such effect are unclear. Most vertical HIV transmission occurs late during pregnancy and advanced maternal HIV infection (as evidenced by high viral load and low CD4 lymphocyte count) has been reported to be a risk factor for mother–to-child transmission [31]. It is therefore possible that any difference in size at birth between HIV-infected and HIV-exposed uninfected infants may be the consequence of effects of the virus on the mother (expressed as HIV related illness) rather than directly on the (infected) fetus [25]. Ryder *et al.* reported that the mean birth weight of infants whose mothers were at an advanced stage was significantly lower than that of infants born to HIV-infected asymptomatic mothers [32]. Moye *et al*. showed that in the USA, the mean birth weight Z-scores of HIV-infected infants was lower than those of HIV-exposed uninfected children; however, they also reported that disease stage as measured by mean prenatal CD4 T lymphocyte count did not appear to influence birth weight Z-score for HIV-exposed, and either HIV-infected or uninfected, infants [33]. HIV staging data were not available in our study, so we are not able to verify this observation. Other authors suggest that the type of ART (particularly the use of protease inhibitors) taken by mothers during pregnancy either for PMTCT or for their own health may explain this relation [34]. However, such drugs are used only as second line regimen, and are therefore not widely taken in Cameroon now.

We found a significant difference in birth weight Z-scores between HIV-exposed uninfected and HIV-unexposed uninfected infants. This result confirms the findings from other studies describing lower anthropometric outcomes among HIV-exposed uninfected than HIV-unexposed uninfected infants [4], [33]. By contrast, other authors found that growth measures for the two groups were similar [3], [16], [18], [19]. The difference observed may be linked to maternal HIV and/or related illnesses or to differences in the distributions of other risk factors between the two infant groups (see Table 1). There are various possible explanations for this difference. First, studies of *in utero* exposure to antiretroviral therapy describe inconsistent results. Studies in Cote d’Ivoire, Ireland and Botswana found that triple therapy during pregnancy was associated with low birth weight or with other particular perinatal outcomes [22]–[24], whereas studies in France and Italy did not find any relationship between ART and birth weight [21], [34]. We did not find any association between the use of any ART (in combinations or as single drugs) during pregnancy by mothers of HIV-exposed uninfected infants with SGAG (aOR = 0.8; 0.1−6.5). Second, exposure of the fetus to maternal HIV and related illnesses and/or to ART may lead to immune system abnormalities [35]; the cause of any such immune abnormality is unclear. Possibly, there is an unusually strong maternal placental response in HIV-infected and ART-treated pregnant women, including substantial placental production of the pro-inflammatory cytokines such as tumor necrosis factor-α and interleukin-8 [36], [37]; which could in association with parasitic infestation (for example malaria) affect fetal growth [24].

We found that primiparity was associated with SGAG. This observation was confirmed by a subgroup analysis of HIV-unexposed uninfected infants and of the HIV-exposed uninfected subgroup. This is consistent with previous reports [7], [17], [38]. In our study, 71 % of primiparous mothers were under 21 years old, corroborating previous findings that primiparity and maternal age are important contributors to fetal growth restriction [39], [40]. Primigravidae are prone to malaria in endemic areas, and placental infections with plasmodium parasites may affect placental function and thereby restrict fetal growth [12].

We also found that ‘any infectious pathology’ during pregnancy was significantly associated with SGAG. Although the evidence is not detailed, other maternal factors such as anemia and malaria, which are frequent among HIV-positive mothers [41] during pregnancy, have been reported to be associated with fetal growth restriction [12], [16], [37], [41].

Neither maternal age nor socioeconomic status (evaluated here as “lack of a functional fridge at home” as a proxy) found in other studies to be associated with SGAG [28] were identified by our study as factors for SGAG. This could be because our study was conducted in three referral hospitals in urban areas where most women were able to afford basic needs.

Our study has several strengths and limitations. One major strengths is that at inclusion, HIV-exposed and HIV-unexposed infants were selected independently of their birth weight, and variables were collected blind to infants’ HIV status. Another is the standardization of birth anthropometric measurements using Z-scores and taking gestational age and gender in consideration; this is more robust than using a single measure alone. We also controlled for many maternal sociodemographic and obstetrical characteristics which could act as potential confounders. The inclusion of a control group of HIV-unexposed uninfected infants is another strength benefit. There are also various limitations: gestational age was not collected with sufficient precision, potentially leading to bias in classification. However, any such bias is likely to affect the two groups of infants in the same manner, minimizing any misclassification bias. Some key confounding factors were not collected, and in particular alcoholism, smoking and nutritional status of mothers, and parental anthropometric measures. These factors have been described to influence infants’ birth weight. They are very important in term of prevention because they represent modifiable risk factors on which action can be directed.

In summary, we report a clear association between maternal HIV infection and SGAG among HIV-exposed uninfected infants. This is a further argument for the need to optimize the provision of care to pregnant HIV-infected women (especially immune-suppressed ones) and their infants. Indeed, monitoring growth and developing strategies to prevent growth faltering in these infants are required. A better evaluation of the consequences on fetal growth of ART during pregnancy would be extremely beneficial. This is now particularly pertinent because the World Health Organization (WHO) is encouraging countries to engage in option B+, which is the widespread alternative access to HAART during pregnancy for the prevention of mother-to-child HIV transmission.
